# P-1982. Negative Predictive Value of Methods to Identify Underlying Medical Conditions with and without Use of a Lookback Period among Adults with a Healthcare Encounter for Acute Respiratory Illness, September 2023 – August 2024

**DOI:** 10.1093/ofid/ofaf695.2149

**Published:** 2026-01-11

**Authors:** Amber Kautz, Morgan Najdowski, Kristin K Dascomb, Sara Y Tartof, Karthik Natarajan, Stephanie Irving, Nicola P Klein, Shaun J Grannis, Toan Ong, Sarah W Ball, Malini B DeSilva, Jennifer DeCuir, Ruth Link-Gelles, Ryan E Wiegand, Amanda B Payne

**Affiliations:** Centers for Disease Control and Prevention, Atlanta, Georgia; CDC, Atlanta, Georgia; Intermountain Healthcare, Murray, Utah; Kaiser Permanente Southern California, Pasedena, CA; Columbia University, New York, New York; Kaiser Permanente Center for Health Research, Portland, Oregon; Division of Research Kaiser Permanente Vaccine Study Center, Oakland, California; Indiana University, Indianapolis, Indiana; University of Colorado Anschutz Medical Campus, Centennial, Colorado; Westat, Newton, Massachusetts; HealthPartners Institute, Bloomington, Minnesota; Centers for Disease Control and Prevention, Atlanta, Georgia; Centers for Disease Control and Prevention, Atlanta, Georgia; Centers for Disease Control and Prevention, Atlanta, Georgia; CDC, Atlanta, Georgia

## Abstract

**Background:**

Vaccine effectiveness (VE) studies are necessary to understand how well vaccines work in the real world. Many VE studies rely on health records to capture underlying medical conditions (UMCs) from a single acute respiratory illness- (ARI) associated encounter, which may bias VE if UMCs are not fully captured. We assessed capture of UMCs from a single acute encounter and a lookback period.

**Figure:**
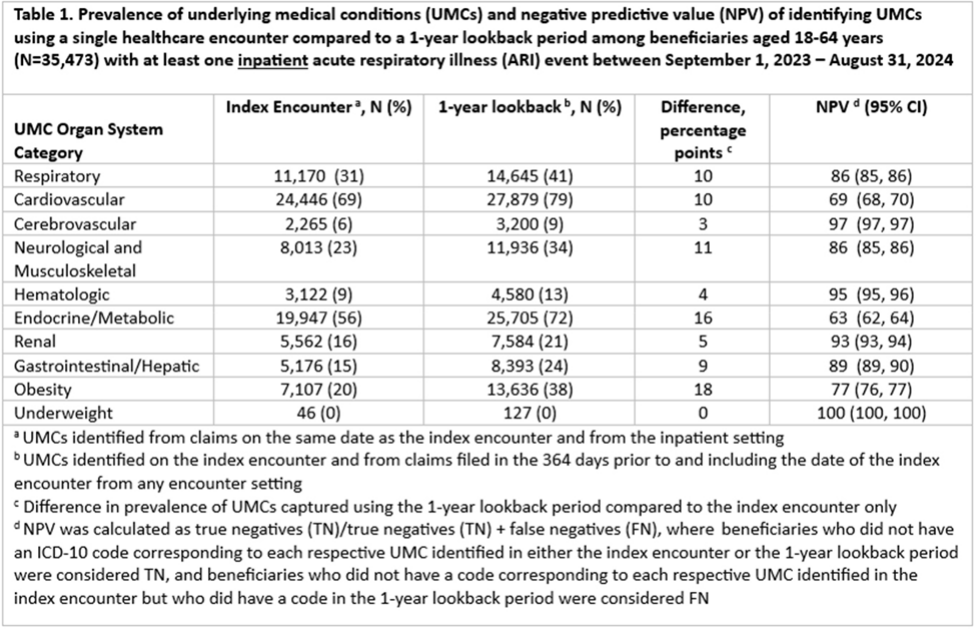

**Figure:**
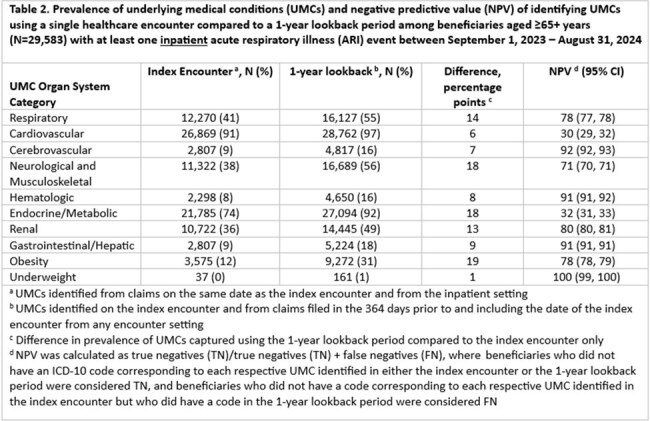

**Methods:**

Data were used from MarketScan® Treatment Pathways, a healthcare claims dataset, between September 1, 2023 – August 31, 2024. We included beneficiaries aged ≥18+ years with ≥1 inpatient or emergency department (ED) claim containing an ICD-10 code for ARI who had 3 years of continuous enrollment in a participating insurance plan prior to the date of their first ARI claim (i.e., index encounter). The prevalence of UMCs was calculated using ICD-10 codes from 1) the index encounter, and 2) the 1-year lookback period; and the difference was reported. Negative predictive value (NPV) with 95% exact binomial confidence intervals was calculated for identification of UMCs on the index encounter date, using the 1-year lookback period to define true negatives. Results were stratified by age group and encounter setting.

**Figure:**
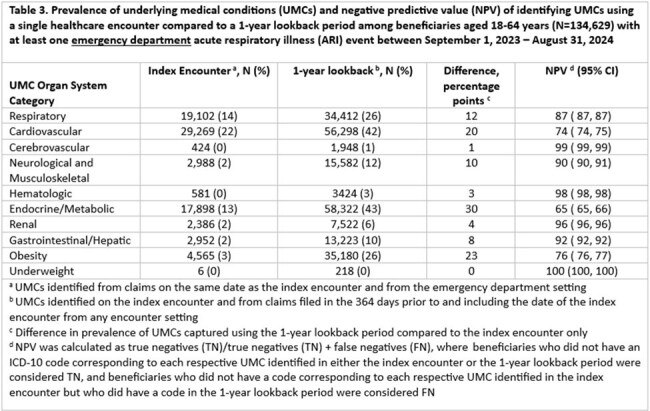

**Figure:**
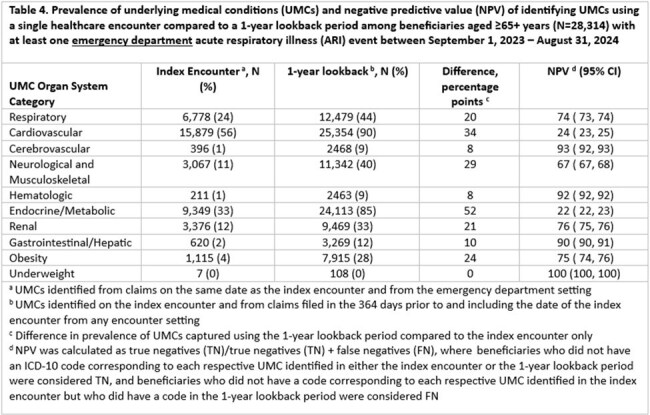

**Results:**

Among 65,056 beneficiaries with ≥1 inpatient ARI event and 162,943 with ≥1 ED ARI event, the most prevalent UMC categories were cardiovascular, endocrine/metabolic, and respiratory (Tables 1-4). Among beneficiaries aged 18–64 years, NPV was < 80% for cardiovascular, endocrine/metabolic, and obesity categories; median difference in prevalence was 9.5 percentage points (pp) (min=0, max=30) (Tables 1 and 3). Among beneficiaries aged ≥65+ years, NPV was ≤80% for respiratory, cardiovascular, neurological and musculoskeletal, endocrine/metabolic, renal, and obesity categories; median difference in prevalence was 13.5 pp (min=0, max=52) (Tables 2 and 4). NPV, regardless of age, was > 90% for UMC categories with the lowest overall prevalence (i.e., cerebrovascular, hematologic, and underweight categories) (Tables 1-4).

**Conclusion:**

NPV was < 80% for common UMCs when identified using a single ARI encounter compared to a 1-year lookback period. Misclassification may influence VE estimates if UMCs are confounders in VE studies.

**Disclosures:**

Sara Y. Tartof, PhD, MPH, Centers for Disease Control and Prevention: Grant/Research Support Karthik Natarajan, PhD, Centers for Disease Control and Prevention: Grant/Research Support Stephanie Irving, MHS, Westat: Grant/Research Support Nicola P. Klein, MD, PhD, AstraZeneca: Grant/Research Support|Centers for Disease Control and Prevention: Grant/Research Support|GlaxoSmithKline: Grant/Research Support|Janssen: Grant/Research Support|Merck: Grant/Research Support|Moderna: Grant/Research Support|Pfizer: Grant/Research Support|Sanofi Pasteur: Grant/Research Support|Seqirus: Grant/Research Support Shaun J. Grannis, MD, MS, Centers for Disease Control and Prevention: Grant/Research Support|National Institutes of Health NCATS: Grant/Research Support|National Institutes of Health NIMH: Grant/Research Support Toan Ong, PhD, Centers for Disease Control and Prevention via Westat: Grant/Research Support|Patent Title: Systems and Methods For Record Linkage: Patent Number: PCT/US2018/047961|PCORI: Travel Support|Regenstrief Institute: Advisor/Consultant|Regenstrief Institute: Travel Support Sarah W. Ball, MPH, ScD, Centers for Disease Control and Prevention, Contract #200-2019-F-06819: Grant/Research Support|Centers for Disease Control and Prevention, Contract #75D30121D12779: Grant/Research Support|Novavax: Grant/Research Support Malini B. DeSilva, MD, MPH, Centers for Disease Control and Prevention Vaccine Safety Datalink: Grant/Research Support|Westat: Grant/Research Support Ryan E. Wiegand, PhD, Merck & Co., Inc.: Stocks/Bonds (Public Company)|Sanofi S.A.: Stocks/Bonds (Public Company)

